# Late dislodgement of left bundle branch pacing lead and failure of left ventricle capture management algorithm

**DOI:** 10.1016/j.ipej.2025.04.004

**Published:** 2025-04-22

**Authors:** Pugazhendhi Vijayaraman

**Affiliations:** Geisinger Heart Institute, Wilkes Barre, PA, USA

**Keywords:** Left bundle branch pacing, Automatic capture management, Late lead dislodgement

## Abstract

A 74-year-old man with nonischemic cardiomyopathy, LV ejection fraction (LVEF) of 20 % and left bundle branch block (LBBB) underwent successful cardiac resynchronization therapy defibrillator (CRT-D) implantation utilizing LBBP lead in the LV port. Despite normalization of LVEF at 6 months, patient presented with late lead dislodgment at 10 months post-implant and decline in LV function. LV capture management algorithm was inactive due to sensing of conducted beat as ventricular fibrillation sensed event. Removal and replacement of LBBP lead resulted in recovery of LV function.

## 1 Introduction

His bundle pacing is the most physiologic form of ventricular pacing [1]. However, left bundle branch pacing (LBBP) has become the preferred approach in most patients due to stable capture thresholds and the ability to pace beyond the site of block [2,3]. Recently studies have demonstrated that LBBP is associated with greater reduction in the composite endpoint of death or heart failure hospitalization compared to biventricular pacing in patients with indications for cardiac resynchronization therapy [4,5]. Automatic capture management (ACM) algorithms are quite reliable and routinely used in pacemakers and cardiac resynchronization therapy devices including right atrial (RA), right ventricular (RV) and left ventricular (LV) leads [6,7]. We report failure of LV ACM to identify lead dislodgement in a patient with LBBP.

## 2 Case presentation

**Fig. 1:**
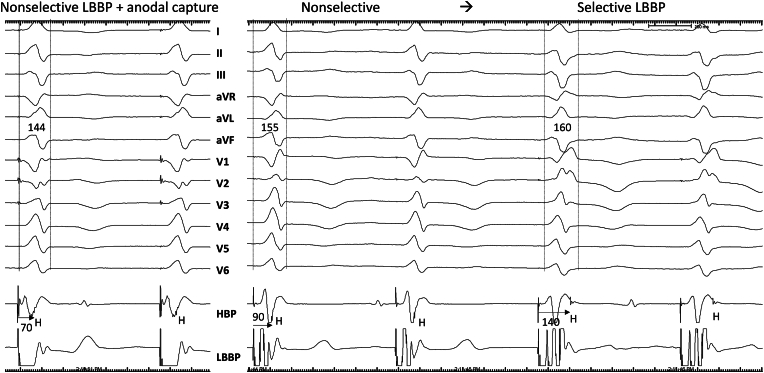
**Left bundle branch capture.** Threshold testing in bipolar mode demonstrates transition from anodal capture + nonselective left bundle branch pacing (LBBP) to nonselective LBBP and subsequent selective LBBP. Note the retrograde His (H) electrograms and prolongation of stimulus to His intervals from 70 ms during anodal capture to 90 ms with LV septal capture to 140 ms during selective LBB capture.

**Fig. 2:**
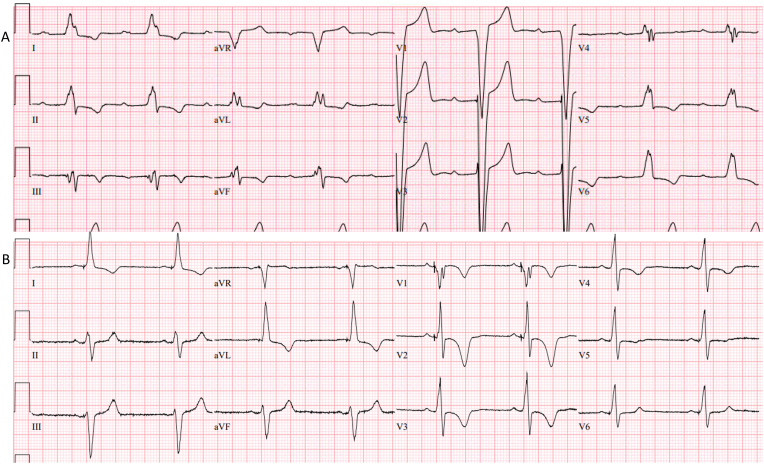
Twelve lead ECG at baseline (A) and following left bundle branch pacing (B).

**Fig. 3:**
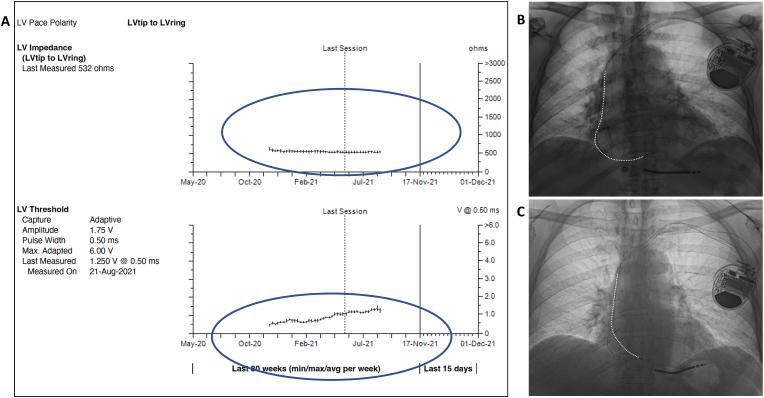
**Device interrogation and chest radiograph. A.** Left ventricular (LV) pacing impedance and capture threshold trends from device interrogation in November 2021 is shown. While the pacing impedance remained stable, the capture threshold appeared to have increased from 1.25V in May 2021 to 1.75V on August 2021, following which no further daily measurements have been performed. **B.** Chest X-ray post device implant shows normal appearing leads with adequate slack on all 3 leads. **C**. CXR one year later, demonstrates dislodgement of the LBBP lead with no slack while the right ventricular and right atrial leads appear stable. While line in figures B and C were drawn on the LBBP lead to better visualize the lead.

**Fig. 4:**
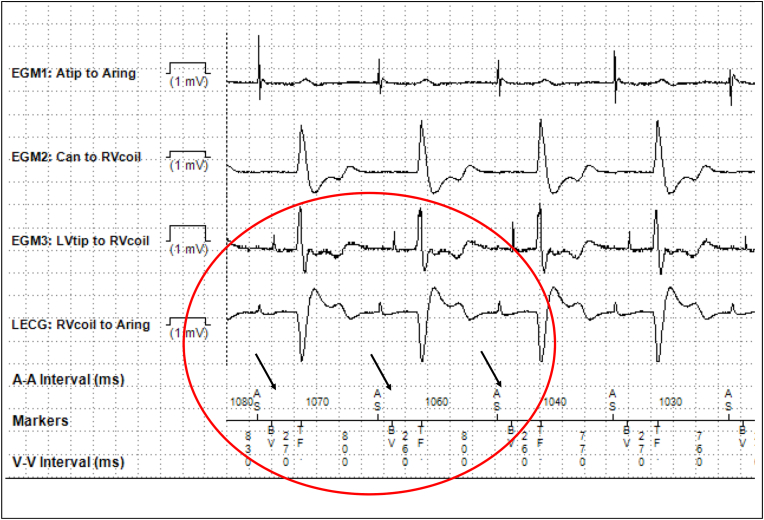
**Device interrogation showing ventricular non-capture**. Electrograms from the device on presentation are shown. Atrial sensed events are followed by biventricular (BV) pacing and ventricular fibrillation (TF) sensed events. Right ventricular (RV) lead was programmed to minimal output (0.5V @ 0.05 ms) to facilitate left ventricle (LV) only pacing. However, due to LV lead dislodgement, there is ventricular non-capture followed by native conduction with long delay and a sensed ventricular (TF, 260–270 ms) event that falls in the ventricular fibrillation (VF) zone. This pattern repeats itself resulting in 20/40 sensed events continuously falling in VF zone.

A 74-year-old man with nonischemic cardiomyopathy, LV ejection fraction (LVEF) of 20 % and left bundle branch block (LBBB) underwent successful cardiac resynchronization therapy defibrillator (CRT-D, Claria MRI, DF4, IS1, Medtronic Inc, Minneapolis, MN) implantation utilizing LBBP lead in the LV port in November 2020. Left bundle branch capture was confirmed by demonstration of nonselective to selective capture transition during threshold testing (Fig. 1). The RV pacing output was programmed to subthreshold (0.5V @ 0.05 ms) to promote bipolar, LV only pacing to fuse with native right bundle branch conduction (Fig. 2). LV ACM was programmed ‘on’ in the LV lead. LBBP lead threshold was stable at 1V at 6-month follow-up and follow-up echocardiogram revealed that LVEF had normalized. During office follow-up at 1 year (November 2021) it was noted that the device had stopped assessing LV capture threshold and impedance in August 2021(Fig. 3A). Presenting rhythm demonstrated ventricular pacing spikes (BV) without capture but conducted beats from sinus rhythm (AS) sensed as ventricular fibrillation (TF) event (Fig. 4). Threshold testing from the LV (LBBP) lead revealed no capture and chest-x-ray demonstrated lead dislodgement into RV (Fig. 3B and C). Repeat echocardiogram showed ventricular dyssynchrony and moderately reduced LVEF to 38 %. LBBP lead was removed by manual traction and replaced with a new LBBP lead. RV pacing output was programmed to 0.5V above capture threshold with LV-RV delay of 80 ms. During 3-year follow-up, the LBBP thresholds remained stable and LVEF had normalized.

## 3 Discussion

We present a case of LBBP in a patient with severe LV dysfunction and LBBB in whom LV function fully recovered at 6 months. We have observed early dislodgement of LBBP lead usually within 24 hours or up to 2 weeks in follow-up. It is unusual to have late dislodgement of LBBP lead especially more than 10 months later when complete recovery of LV function has occurred as in this case. The mechanism for this late dislodgement was not fully understood. Potential explanations include: 1. Twiddler syndrome – manipulation of the device in the pocket; however, both the atrial and the ventricular ICD leads remained unchanged; 2. Recovery of LV function with hypercontractility of the septal myocardium leading to potential lead migration; 3. Ratcheting [8] – even though there was adequate slack of the LBBP lead at implant, there is no slack in the LBBP lead in the follow-up x-ray (Fig. 2) and all excess lead appears to be behind the device. This mechanism appeared to be the most likely cause for late lead dislodgement.

Additionally in this case, loss of LV capture was not recognized by the ACM algorithm. LV ACM paces LV for 4 beats and measures the LV pace to RV sense interval. This is the paced interventricular conduction interval. Then, LV ACM paces DDD with a longer AV delay to determine the AP to RV sense interval. If there is no RV sense, the device will provide biventricular pacing. This is the AV conduction interval. The device determines the difference between the two intervals. If the difference in the two conduction intervals is ≥ 80 ms, LV ACM will proceed. LV Capture occurs if the RV sense is due to interventricular conduction. LV non-capture occurs if the RV sense is due to AV conduction. If the patient is dependent, a dropped beat may occur when the LV pace does not capture during the threshold test. LV ACM test attempts to run nightly at 1:00 a.m. (non-programmable). If any of the following conditions are met, the test will be postponed for 30 minutes: 1. Patient's heart rate is > 90 bpm; 2. Patient's rhythm is irregular; 3. A ventricular tachycardia/ventricular fibrillation episode has occurred recently.

In this case, LV ACM was not attempted by the device due to the ongoing AS-BV-TF resulting in tachycardia detection count of 20/40. When the tachycardia detection count is 3 or greater (or has actually detected an episode) the device will suspend lower priority functions including, but not limited to, capture management and impedance measurements [2]. This failure of LV ACM to detect loss of capture from late dislodgement of the LBBP lead occurred due to loss of LV capture, subthreshold RV output, and long native AV conduction resulting in sensed event outside the blanking period. LBBP lead was successfully replaced. This scenario is less likely to occur if the currently available LV only pacing programmable feature is used. However, in dependent patients it is preferable to use LV-RV delay of 80 ms with RV output programmed above capture threshold. This case highlights the unique challenge in this patient with BiV ICD and a LBBP lead in the left ventricular (LV) port who was programmed to subthreshold right ventricular (RV) output to promote LBBP only with RV fusion.

## 4 Conclusions

Late dislodgement of LBBP lead is a rare complication. LV ACM algorithm may not provide early warning of LBBP (or LV) lead dislodgement.

## Disclosures

PV -Speaker, Consultant, Research, Fellowship support – Medtronic; Consultant – Abbott, Biotronik, Boston Scientific; Patent- HBP delivery tool.

## Funding

None.

## Declaration of competing interest

The authors declare the following financial interests/personal relationships which may be considered as potential competing interests: Pugazhendhi Vijayaraman reports a relationship with Medtronic that includes: consulting or advisory, funding grants, speaking and lecture fees, and travel reimbursement. Pugazhendhi Vijayaraman reports a relationship with BIOTRONIK Inc that includes: consulting or advisory and speaking and lecture fees. Pugazhendhi Vijayaraman reports a relationship with Boston Scientific Corporation that includes: consulting or advisory and speaking and lecture fees. Pugazhendhi Vijayaraman reports a relationship with Abbott that includes: consulting or advisory and speaking and lecture fees. Pugazhendhi Vijayaraman has patent HBP delivery tool pending to Pugazhendhi Vijayaraman. If there are other authors, they declare that they have no known competing financial interests or personal relationships that could have appeared to influence the work reported in this paper.
